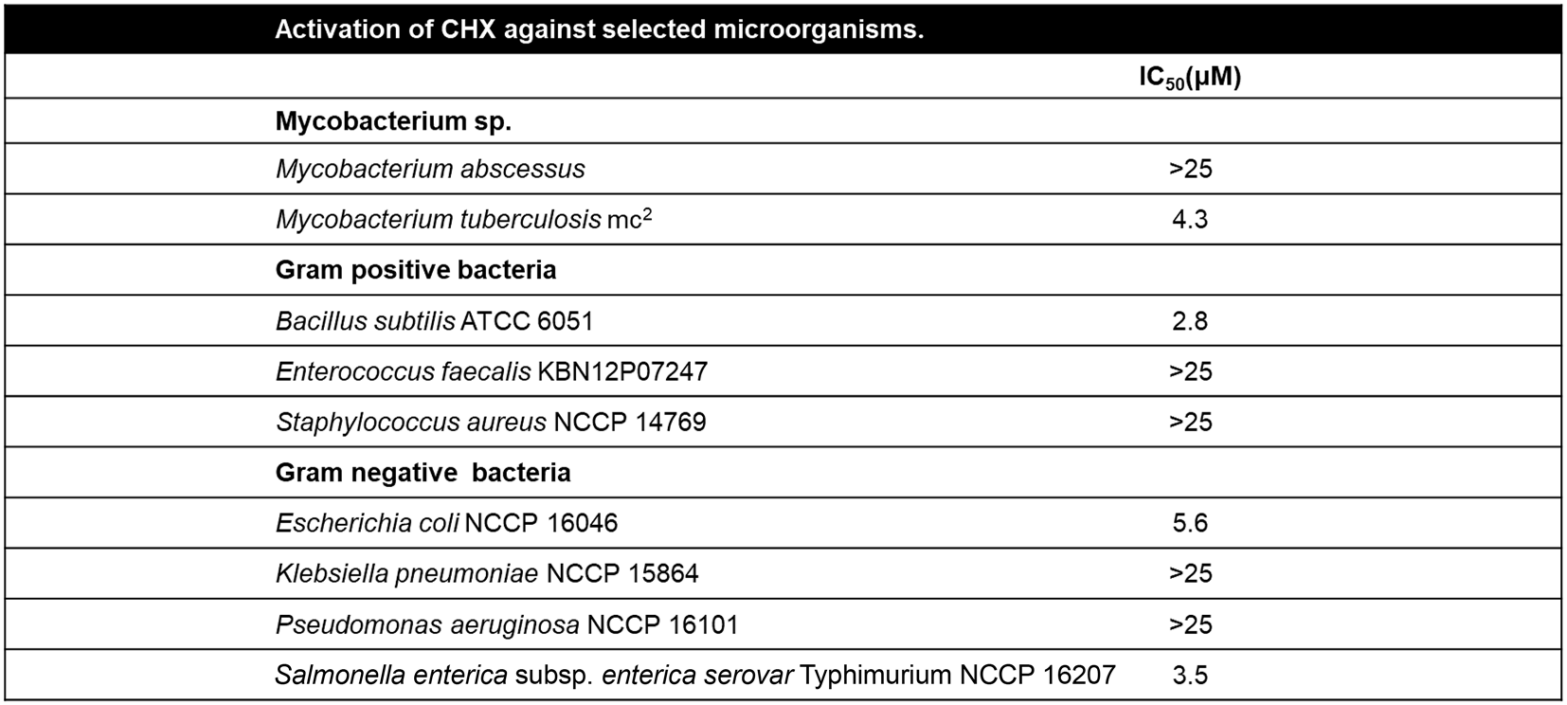# Identification of a Drug Candidate against *Mycobacterium avium* Using Pandemic Response Box

**DOI:** 10.4014/jmb.2506.06006

**Published:** 2025-08-18

**Authors:** Seunghyeon Jeon, Yubin Lee, Jihyeon Yun, Bo Eun Heo, Anwesha Ash, Cheol Moon, Chul-Su Yang, Jichan Jang

**Affiliations:** 1 Division of Life Science, Department of Bio & Medical Big Data (BK21 Four Program), Research Institute of Life Science, Gyeongsang National University, Jinju 52828, Republic of Korea; 2 Department of Molecular & Life Science and Department of Medicinal and Life Science, Hanyang University, Ansan 15588, Republic of Korea; 3 Department of Clinical Laboratory Science, Semyung University, Jecheon 27136, Republic of Korea

**Keywords:** *Mycobacterium avium* complex (MAC), nontuberculous mycobacteria (NTM), extrapulmonary infections, clorhexidine (CHX), alexidine (AX), skin infections

## Abstract

MAC (*Mycobacterium avium* complex) is a naturally occurring environmental microorganism found worldwide in sources such as soil and water. Among nontuberculous mycobacteria (NTM), MAC is the species most commonly responsible for pulmonary infections, particularly in immunocompromised individuals. In addition to pulmonary disease, extrapulmonary *M. avium* infections can present as disseminated, cutaneous, or lymphatic diseases. Skin infections caused by *M. avium* can vary significantly between patients, with both localized and disseminated forms observed. Despite the increasing prevalence of extrapulmonary NTM infections, treatment outcomes remain suboptimal, underscoring the need for novel therapeutic strategies. In this study, we conducted in vitro dual-screening using the Pandemic Response Box against *M. avium* 104, and identified alexidine (AX) as a promising candidate for therapy. Further evaluation demonstrated that chlorhexidine (CHX), a structurally distinct bis-biguanide compound, also exhibited potent inhibitory activity against *M. avium* growth in vitro, as well as in a zebrafish model of *M. avium* infection and treatment. These findings suggest that CHX may be a potential therapeutic candidate for treating *M. avium* skin infections.

## Introduction

*Mycobacterium avium*, a prominent species within the *Mycobacterium avium* complex (MAC), was first isolated in 1890 from a chicken with cavitary disease resembling tuberculosis, although human infections were not documented until 1930 [1]. This species comprises four diverse subspecies: *M. avium* subsp. *avium*, *M. avium* subsp. *paratuberculosis*, *M. avium* subsp. *silvaticum*, and *M. avium* subsp. *hominissuis* [2]. MAC organisms inhabit environments common to humans, such as soil and water, facilitating aerosol transmission. Notably, environmental reservoirs include household plumbing systems and hospital water supplies, increasing exposure risk through inhalation of aerosolized water from plumbing fixtures and showers, treatment plants, and distribution networks.

MAC exhibits resilience and resistance to harsh conditions, including chlorine disinfectants, desiccation, low nutrient availability, and hypoxic environments. It forms robust biofilms and can survive elevated temperatures typical of water heaters [3-6]. Consequently, human exposure to MAC is widespread. While infected individuals are often asymptomatic, MAC affects vulnerable populations, including those with underlying lung diseases such as bronchiectasis and chronic obstructive pulmonary disease (COPD), as well as immunocompromised patients due to autoimmune disorders or immunosuppressive therapies [7]. Postmenopausal women and individuals over 65 years old also show increased susceptibility to MAC lung disease [8].

MAC accounts for approximately 80% of nontuberculous mycobacterial (NTM) pulmonary disease cases globally, with significant prevalence reported in countries such as Ireland, South Korea, and the United States [9, 10]. Extrapulmonary MAC infections manifest as disseminated, cutaneous, or lymphatic disease. Disseminated MAC primarily affects patients with acquired immunodeficiency syndrome, inherited immunodeficiencies, autoimmune diseases, leukemia, or those undergoing iatrogenic immunosuppression [11]. Treatment outcomes remain suboptimal due to factors including pathogen variability, patient immune status, and disease severity [12]. Current guidelines recommend a combination regimen of a macrolide (azithromycin or clarithromycin), ethambutol, and a rifamycin (rifampin or rifabutin) [13]. However, the sufficiency of this three-drug regimen and optimal treatment duration, which typically lasts 6 to 12 months, remain uncertain [9,14].

Given the challenges of prolonged therapy, drug toxicity, and emerging macrolide resistance, there is an urgent need to develop safer and more effective treatment options. Despite this, drug discovery efforts targeting MAC are limited [15]. Compared to *Mycobacterium tuberculosis*, the MAC drug development pipeline is notably under-developed and only underscores the critical need for novel anti-MAC agents. NTMs, including MAC, generally exhibit resistance to most anti-tubercular drugs and possess distinct susceptibility and resistance profiles from *M. tuberculosis*. Furthermore, no NTM-specific candidates are currently advancing in preclinical development [16].

In this study, we screened the Pandemic Response Box, a library of 400 drug-like compounds curated by Medicines for Malaria Venture (MMV) with known activity against a range of neglected diseases, including ascariasis, Buruli ulcer, Chagas disease, and malaria [17]. These compounds were pre-evaluated for cytotoxicity and exhibit profiles suitable for early-stage drug discovery. Our screening against *M. avium* identified alexidine (AX) as the most potent hit. In addition, chlorhexidine (CHX), a structurally related bis-biguanide compound, also showed promising activity and may represent a potential therapeutic candidate for *M. avium* skin infections.

## Materials and Methods

### Bacterial Strains and Culture Conditions

For experiments in this study, *Mycobacterium avium* subsp. *hominissuis* (*M. avium* 104) and *Mycobacterium avium* subsp. *avium* Chester ATCC 700898 (*M. avium* 101) were used. Clinical isolates were purchased from the Korea *Mycobacterium* Resource Center (KMRC, Republic of Korea). *Mycobacterium abscessus* CIP104536^T^ was kindly provided by Dr. Laurent Kremer (CNRS, IRIM, Université de Montpellier, France), and *Mycobacterium tuberculosis* mc² by Dr. William R. Jacobs, Jr. (Department of Microbiology and Immunology, Albert Einstein College of Medicine, USA). *Bacillus subtilis* was obtained from the American Type Culture Collection (ATCC), and *Enterococcus faecalis* was provided by the Korea Biobank Network (KBN). All other pathogenic bacterial strains were obtained from the Fastidious Specialized Pathogen Resources Bank, a member of the National Culture Collection for Pathogens, supported by the Ministry of Health and Welfare, Republic of Korea.

All *M. avium* strains were grown in Middlebrook 7H9 broth (BD Biosciences, USA) supplemented with 10%(*v:v*) albumin, dextrose, and catalase (ADC; BD), 0.2% (*v:v*) glycerol, and 0.05% (*v:v*) Tween 80 (7H9^G/T/ADC^) or Middlebrook 7H10 agar plates (BD) containing 10% (*v:v*) oleic acid and ADC (OADC; BD) enrichment and 0.5%(*v:v*) glycerol (7H10^G/T/OADC^). All cultures were grown at 37°C with shaking at 180 rpm. Clarithromycin (CLA), amikacin (AMK), alexidine (AX) and chlorhexidine (CHX) were purchased from Sigma-Aldrich (USA). The activity of CHX was evaluated in cation-adjusted Mueller–Hinton broth, as described previously [18].

### MMV Library Screening

MMV Pandemic Response Box was initially screened against *M. avium* 104 strain at 20 μM in 96-well plates in 7H9^G/T/ADC^ medium using REMA (see “REMA” below). The culture was grown to log phase (OD_600_ 0.4–0.8) and diluted to OD_600_ 0.005. Then, 98 μl of culture was mixed with 2 μl of compounds, and plates were sealed with self-adhesive films and incubated at 37°C for 4 days. The plates had a column of 2% DMSO as negative control (drug-free) and CLA as positive control. Growth inhibition rate compared to control groups was plotted using GraphPad Prism version 6 (GraphPad Software, Inc., USA). Compounds that had a >80% inhibitory effect at 20 μM were classified as primary hits. Libraries were screened again at 5 μM, as performed for the primary screen, and compounds that had a >80% inhibitory effect in common with primary hits were classified as confirmed hits.

### Resazurin Microtiter Assay (REMA) and Determination of Minimum Inhibitory Concentration (MIC)

The MIC for each compound was determined using a resazurin reduction microtiter assay (REMA) as previously described [19]. Briefly, bacterial culture was grown to log phase and diluted to OD_600_ 0.005 in 7H9^G/T/ADC^ medium per well, and 50 μl of serial 2-fold dilutions of test compound solution was added to each well of a sterile, polystyrene 96-well cell culture plate (SPL; Republic of Korea). A drug-free growth control was included in each plate to a final volume of 100 μl, and peripheral wells were filled with 200 ml of sterilized water to prevent evaporation during incubation. Plates were incubated at 37°C for 4 days. After 4 days of incubation, 40 ml of resazurin solution was added to the wells as a 0.025% (*w/v*) solution in sterile distilled water using resazurin sodium salt powder (Sigma, USA). Fluorescence was measured by excitation at 560 nm and emission at 590 nm using a SpectraMax M3 multi-mode microplate reader (Molecular Devices, USA). A dose-response curve was constructed using RFU values relative to drug-free conditions. The bacterial growth-related MIC values were determined by 50% (MIC_50_) and 90% (MIC_90_) using GraphPad Prism v6 software.

### Determination of Bactericidal/Static Activity

For the bactericidal/static activity determination assay, *M. avium* 104 was tested. Following three days of incubating compounds at 37°C, the entire contents of the 96-well microtiter plate corresponding to the two-fold diluted compound concentrations (5× and 10× MIC) were plated, and the colony-forming unit (CFU) counts were determined. The Minimum Bactericidal Concentration (MBC) of compounds against the *M. avium* 104 was established as the lowest drug concentration necessary to induce ≥99.9% cell death compared to the untreated control at the 0-h time point. Bactericidal antibiotics typically exhibit an MBC value of ≤4 times the MIC, while bacteriostatic antibiotics generally have an MBC >4 times the MIC.

### Antibiotic Killing Kinetics In Vitro

The time-kill kinetics assay and an agar plate-based method were used to evaluate the bactericidal activity of AX and CHX. Briefly, *M. avium* 104 was inoculated at 1.5 × 10^6^ CFU/ml into 7H9 broth supplemented with varying concentrations (0, 5×, 10×, and 20× MIC) of AX or CHX. CLA was included as a positive control for growth inhibition, while DMSO served as the vehicle control. Bacterial viability was assessed at 24-h intervals over a 6-day period by plating serial dilutions onto Middlebrook 7H10 agar supplemented with OADC, followed by enumeration of CFUs.

### Isolation of Resistant Mutants

*M. avium* 104 was cultured to log (exponential) phase. Then, 250 μl of OD_600_ 1.0 (approx. 9 × 10^7^ CFU/ml) was plated on 7H10^G/T/OADC^ medium containing 1×, 5× and 10× MIC_90_ of the compound of interest. The MIC_90_ values on the medium used in this experiment were 1.89 μM for AX, 1.72 μM for CHX, and 1 μM for AMK as control. The plates were incubated for 4 days in a 37°C incubator. The number of resistant colonies was counted to obtain mutation frequencies. Laboratory-generated resistant mutants were transferred into 7H9^G/T/ADC^ medium as well to conduct the REMA assay. The MIC value was determined for the mutant compared with wild-type strain.

### ZF Infection and Drug Efficacy Assessment in *M. avium*-Infected ZF

All zebrafish (ZF) experiments were approved by the Animal-Research Ethics Committee of Gyeongsang National University (GNU-220710-E0078). *Mycobacterium avium* 104 strain was used for the ZF microinjection experiment. ZF embryos at 30 to 48 h postfertilization were dechorionated and anesthetized with 270 mg/l tricaine at room temperature. Around 3 nL of *M. avium* (400 CFU) in 0.085% phenol red was injected via the caudal veins of ZF larvae using a digital microinjector (MINJ-D; Tritech Research, Los Angeles, CA, USA). The infected ZF were transferred into 96-well plates (2 fish per well containing 200 μl of fish water), before being exposed to each drug. To monitor the survival of the embryos, the compounds were renewed daily, and the plates were incubated at 28.5°C. CHX at final concentrations of 0.025, 0.05, and 0.1 μM was added directly into the blue fish water (using methylene blue 300 μl/l). The efficacy of CHX was compared with CLA control at a concentration of 50 μM. Untreated ZF was used as a negative control. The in vivo efficacy of compounds was determined by bacterial burden counts and the embryo survival kinetics. For quantification of the bacterial burden, 20 infected ZF (5dpi) per group were collected and homogenized, respectively, in 2% Triton X-100 phosphate-buffered saline with Tween 20 (PBS^T^) using a handheld homogenizer (D1000; Benchmark Scientific, USA). After a 10-fold serial dilution, the suspensions were plated onto 7H10^G/T/OADC^ agar plates containing BBL MGIT PANTA (polymyxin B, amphotericin B, nalidixic acid, trimethoprim, and azlocillin; Becton, USA) and then incubated for 4-5days at 37°C to enumerate the number of CFUs. For Kaplan-Meier survival curves, the number of dead ZF was recorded daily until day 13. The CFU quantification and the survival curves were plotted with GraphPad Prism v6 using the Kaplan-Meier curve and the log-rank (Mantel-Cox) test, respectively, to compare the difference between the DMSO control and the treated ZF.

## Results

### Pandemic Response Box Composition and Drug Screening against *M. avium*

**Fig. 1:**
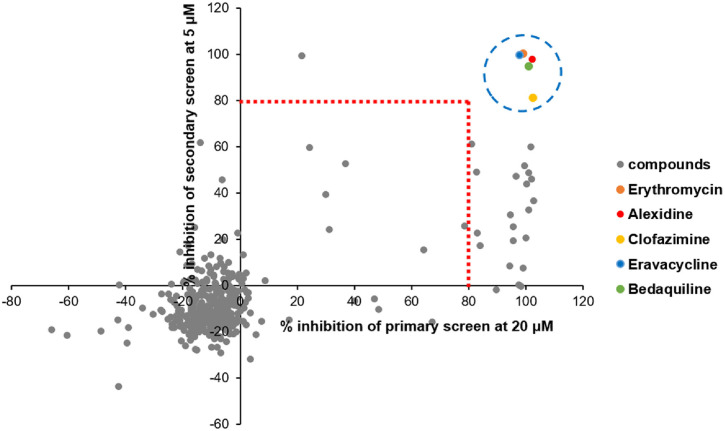
Screening of MMV Open Pandemic Response Box. The primary screen was conducted at 20 μM, followed by a secondary screen at a concentration of 5 μM against *M. avium*. Two independent sets of results were illustrated as scatter plot distributions, identifying five common hits.

**Fig. 2:**
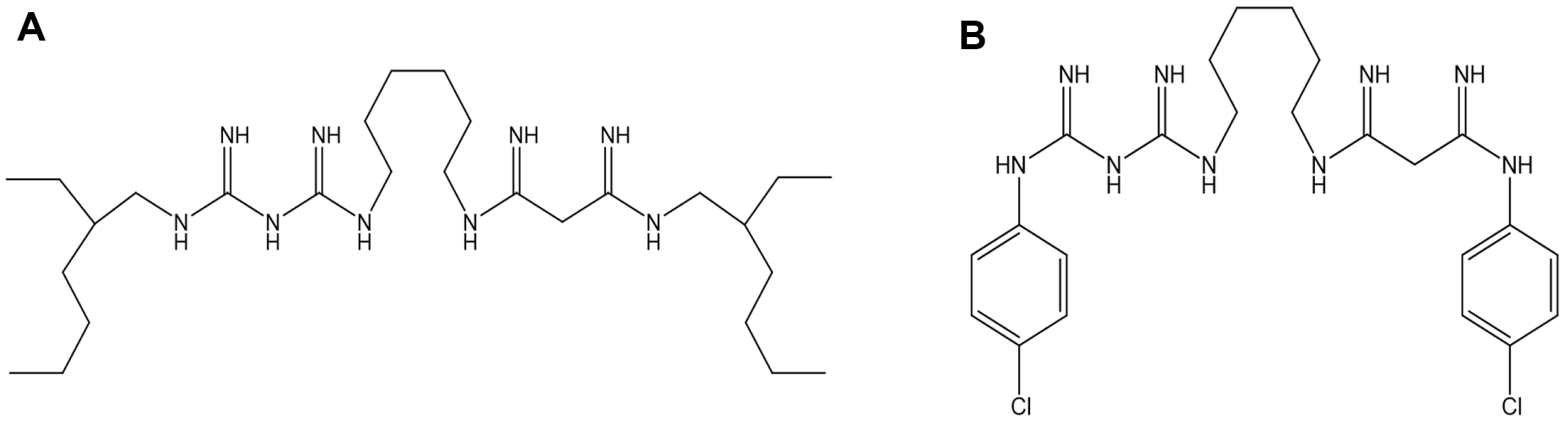
Chemical formula of AX (A) and CHX (B). AX was identified as an active hit during the Pandemic Response Box screen, and its analogue, CHX, was also utilized for further study.

Pandemic Response Box, available free of charge, comprises 400 diverse, drug-like molecules active against neglected diseases. These compounds are supplied in 96-well plates, with each well containing 10 μl of a 10 mM dimethyl sulfoxide (DMSO) solution of the respective compound. For the screening process, the library was acquired in plates from the Medicines for Malaria Venture (MMV) in Geneva, Switzerland. Subsequently, it was diluted to 2 mM and directly transferred into assay plates to achieve a primary screening concentration of 20 μM (resulting in a final concentration of 1% DMSO). An 80% growth inhibition was listed as a primary hit criterion (Fig. 1). This single-point screen identified 21 compounds (primary hit rate 5.3%) that exhibited over 80%inhibitory effects against *M. avium* 104 in 7H9 medium. To include more active compounds for *M. avium* 104, we screened the Pandemic Response Box against *M. avium* 104 at a fixed concentration of 5 μM as secondary screen. The secondary screen runs narrowed down 5 compounds that inhibited the growth of *M. avium* 104 at 5 μM (Fig. 1). The hit rate of 1.25% was notably lower than that typically observed in primary screens. Among the five hits identified, four were previously known to exhibit antimycobacterial activity, prompting us to focus more on alexidine (hereafter referred to as AX), which has not yet been reported as an effective agent against *M. avium* (Fig. 2A). Based on structural and mechanistic similarities described in the literature, we also included chlorhexidine (CHX) in subsequent analyses. Although structurally distinct from AX, CHX is likewise a bis-biguanide compound, and its known antimicrobial properties warranted evaluation of its activity against *M. avium* (Fig. 2B).

### Assessment of AX and CHX Against *M. avium* Strains: Drug Susceptibility and Minimum Bactericidal Concentration Testing

**Fig. 3:**
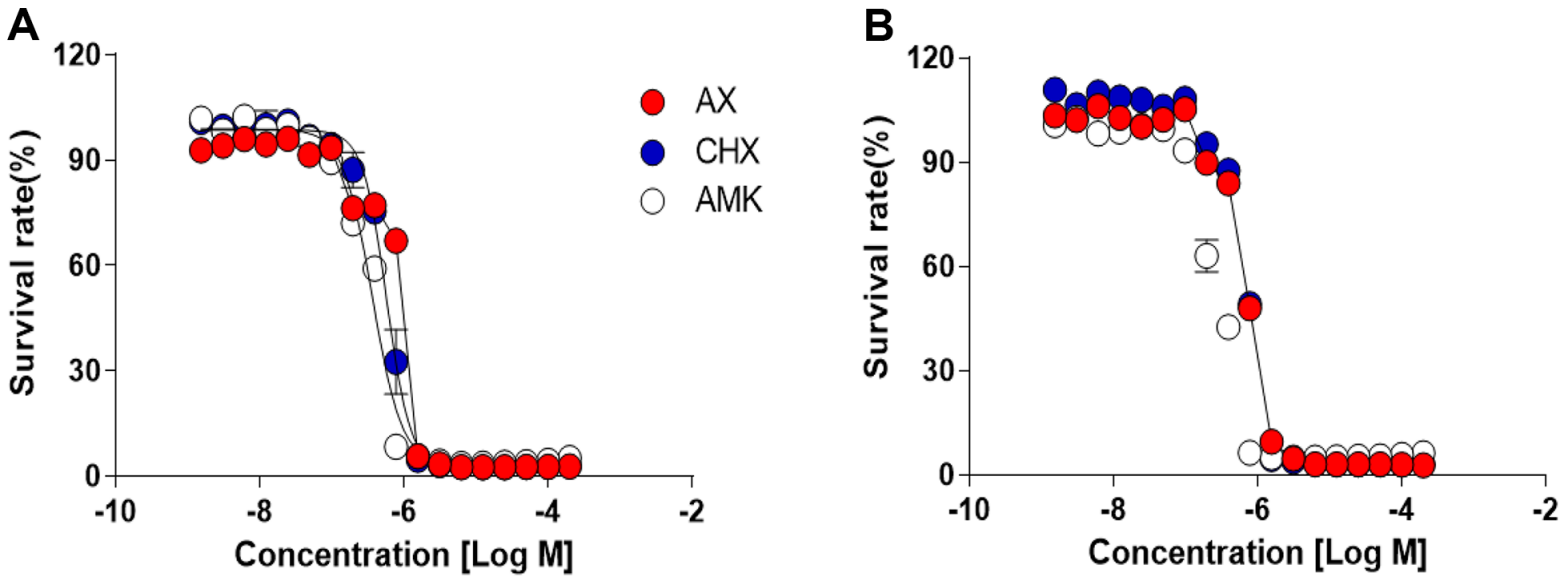
Dose-response curves (DRC) for AX and CHX. Drug susceptibility tests against two different strains of *M. avium* were performed: *M. avium* 104 (**A**) and *M. avium* 101 (**B**), respectively. AMK was employed as the reference drug. Graph fitting is representative of three independent assays, performed in triplicate.

The activities of AX and CHX were confirmed via dose-response curve determination to establish their MIC using the REMA. Their half-inhibitory concentrations (MIC_50_), requiring 50% growth inhibition of *M. avium*, were below 1 μM and similar to that of reference compound AMK, which is considered a major drug in the treatment of MAC (Fig. 3A). Moreover, another virulent strain of *M. avium*, *M. avium* 101, isolated from a patient with Acquired Immune Deficiency Syndrome (AIDS), also displayed bacterial growth inhibition upon treatment with AX and CHX. The MIC_50_ value for *M. avium* 101 after treatment with AX and CHX was very similar to the results observed in *M. avium* 104 (Fig. 3B). In addition, we conducted MIC testing to assess the inhibitory potential of AX and CHX against four different clinical isolates obtained from various sources via the Korean *Mycobacteria*l Resource Center (KMRC). As illustrated in Table 1, AX and CHX treatment notably and dose-dependently suppressed the growth of *M. avium* clinical isolates. For instance, *M. avium* KMRC 00136-41011 and *M. avium* KMRC 00136-41014 exhibited growth inhibition at MIC_90_ values of 1 μM and 2.1 μM for AX, respectively. Similarly, CHX demonstrated MIC_90_ values ranging from 0.8 to 0.9 μM for clinical isolates. The reference compound AMK demonstrated activity comparable to that of AX and CHX against *M. avium* clinical strains. Both AX and CHX exhibited MBC values ranging from 0.8 to 3.1 μM for clinical isolates. In accordance with the definition of bactericidal agents, where the MBC is typically less than four times higher than the MIC, both compounds displayed bactericidal activity against all evaluated *M. avium* isolates.

### Time-Kill Kinetics of AX and CHX against *M. avium*

**Fig. 4:**
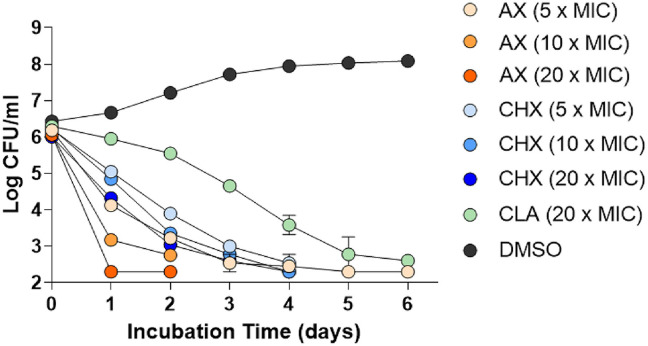
Time-kill kinetics of AX and CHX against *M. avium* 104. *M. avium* 104 was treated with alexidine (AX) or chlorhexidine (CHX) at 5×, 10×, and 20× the minimum inhibitory concentration (MIC), along with clarithromycin (CLA, 20× MIC) as a positive control and DMSO as a negative control. Bacterial viability was assessed by determining colony-forming units (CFU) per milliliter over a 6-day incubation period. AX demonstrated more rapid bactericidal activity than CHX in a concentration-dependent manner. Data represent mean values ± SD from at least three independent experiments.

To further characterize the bactericidal dynamics of AX and CHX, time-kill assays were conducted against *M. avium* 104 at concentrations corresponding to 5×, 10×, and 20× their respective MIC_50_ values as determined by REMA. CLA was included as a positive control. Both AX and CHX exhibited clear bactericidal activity in a concentration- and time-dependent manner (Fig. 4). Notably, AX exhibited rapid bactericidal activity at elevated concentrations. Complete sterilization, with no detectable CFUs, was observed as early as day 1 following treatment with 20× MIC_50_. At 5× MIC_50_, AX resulted in a substantial reduction in CFUs beginning on day 2, with complete inhibition of bacterial growth achieved by day 4. CHX also demonstrated potent bactericidal activity, though with a slower onset compared to AX. At 20× MIC_50_, CHX significantly reduced CFU counts over time, with complete inhibition of bacterial growth achieved by day 4. Lower concentrations (5× and 10× MIC_50_) progressively suppressed bacterial growth, but sterilization required longer exposure times. As shown in Fig. 4, bacterial regrowth was not observed beyond day 4 at any concentration tested, indicating sustained bactericidal effects.

### Drug Resistance Mutant Frequency Comparison between AX and CHX

In the progression of candidate compounds toward therapeutic agents, both antimicrobial efficacy and the potential for resistance development are critical considerations. Therefore, we assessed the emergence of resistant strains of *M. avium* following exposure to high concentrations of AX and CHX and compared their resistance profiles with that of the WT strain. As shown in the supplementary figure and table, CHX-resistant strains exhibited a 3.4- to 5.4-fold increase in MIC values compared to the WT strain, whereas AX-resistant strains showed a markedly higher increase, with MIC values rising 10- to 17.6-fold. To compare the rates of *M. avium* resistance to AX and CHX, we evaluated mutant frequencies resistant to AX and CHX at various concentrations. The *M. avium* 104 culture was plated on Middlebrook 7H10-OADC containing AX and CHX (at 5× and 10× MIC_50_ concentrations). The mutation frequencies of AX-resistant *M. avium* 104 isolates ranged from 1.2×10^−6^ to 4.6×10^−6^. For CHX, mutant frequencies were observed to range from 8.9×10^−8^ to 8.9×10^−8^, whereas the mutation rate of AMK-resistant *M. avium* 104 isolates ranged from 1.6×10^−5^ to 2.2×10^−7^ (Table 2). The mutation frequency of CHX was 13.4 times lower than the range observed for AX. Moreover, both AX and CHX exhibited significantly lower mutant frequencies compared to AMK.

### *In Vivo* Drug Toxicity Testing and *In Vivo* Efficacy Using ZF

**Fig. 5:**
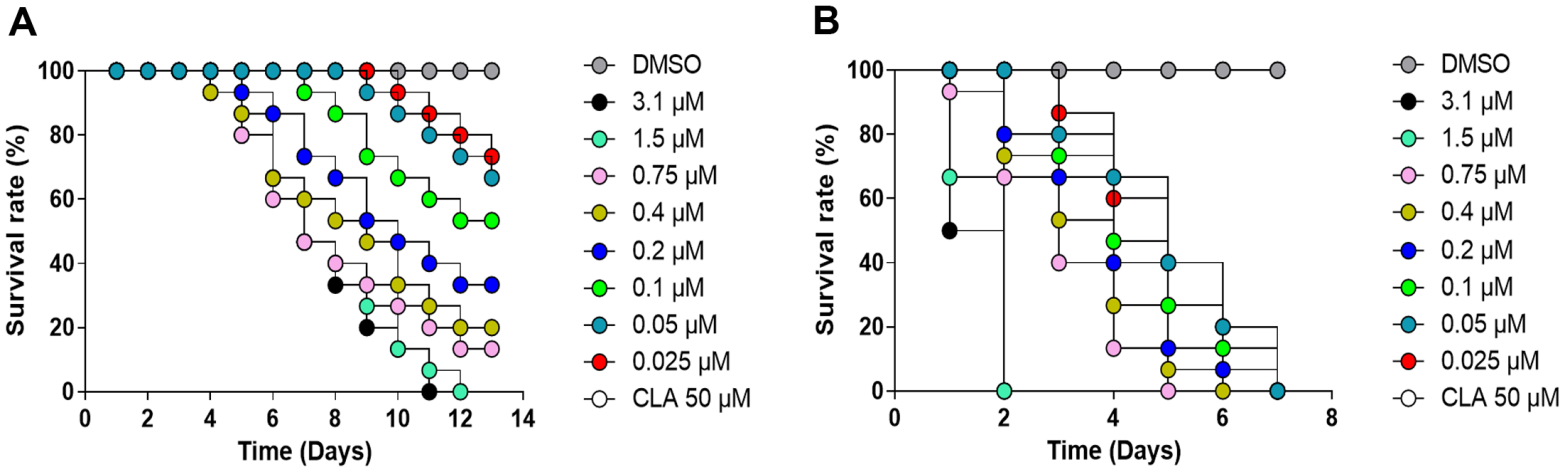
Toxicity of CHX and AX against ZF embryos. The maximum tolerated dose (MTD) was determined using different concentrations of CHX (**A**) and AX (**B**). The ZF survival was followed for 13 dpi. DMSO was used as a vehicle control. Survival rates were plotted as Kaplan-Meier curves using GraphPad Prism software. Statistical differences between groups were analyzed using the log-rank (Mantel-Cox) test. Data represent cumulative results from three independent experiments, with at least 20 embryos per group per experiment.

To further evaluate the in vivo efficacy of hits, we utilized the ZF infection model, a well-established and tractable system for assessing antimicrobial activity. To determine the maximum-tolerated dose (MTD) of AX and CHX in ZF, we administered escalating doses of each compound. Non-infected ZF were treated with a range of concentrations, applying a two-fold dilution series for each compound. As shown in Fig. 5A, CHX-treated ZF did not show reduced survival rates until 3 days after treatment. However, on the 13th day of drug treatment, it was observed that 47% of the ZF treated with CHX at a concentration of 0.1 uM had died. It was noted that as the concentration increased above 0.1 μM, the mortality of CHX-treated ZF increased. For instance, ZF treated with 3.1 μM CHX exhibited 100% mortality within just 11 days of treatment. This finding indicates that CHX concentrations of 0.1 μM or lower are appropriate for future studies using the ZF-*M. avium* infection and treatment model. In contrast, ZF treated with AX exhibited complete mortality after only 7 days of drug treatment at a minimum concentration of 0.025 μM (Fig. 5B). These findings indicate that CHX exhibits lower toxicity than AX in the zebrafish model. Accordingly, subsequent in vivo efficacy studies using the *M. avium*-infected zebrafish model were conducted with CHX alone.

**Fig. 6:**
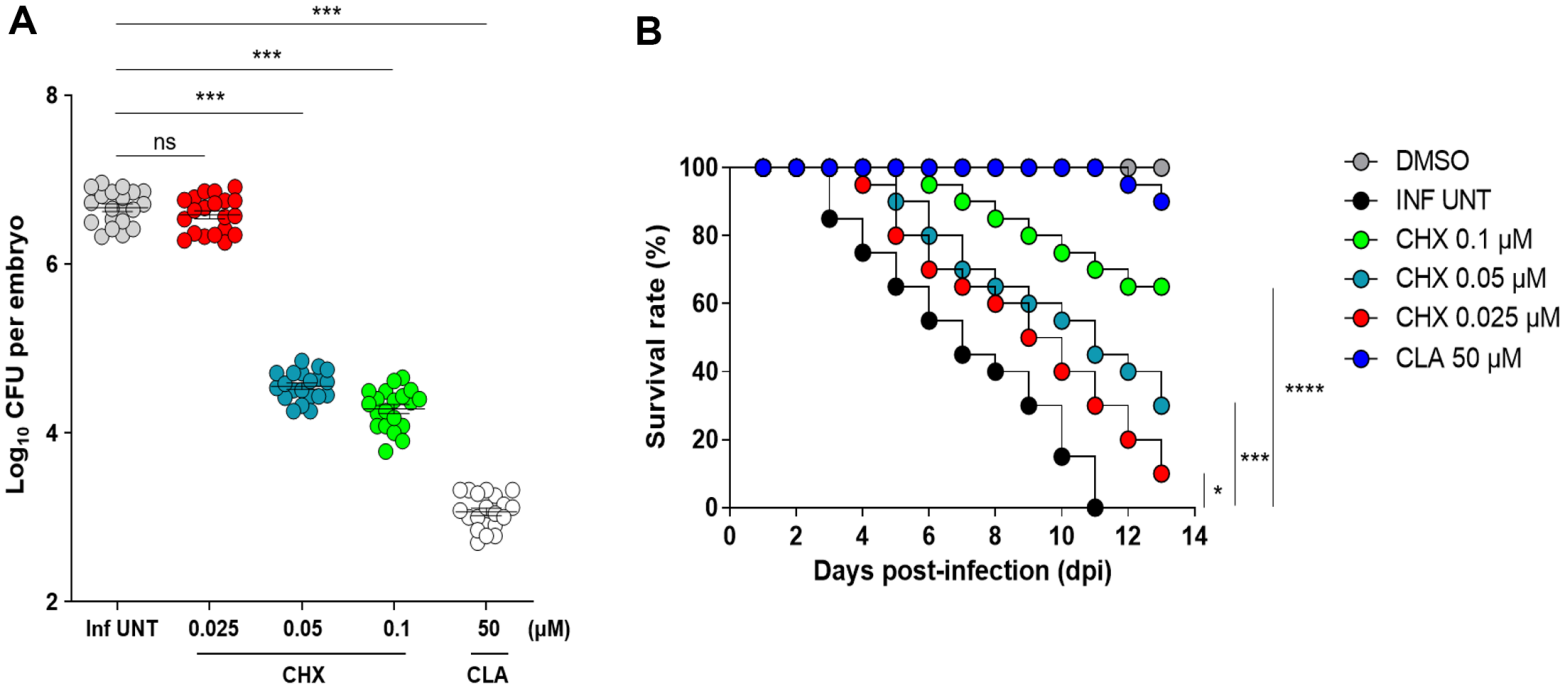
*In vivo* evaluation of CHX activity against *M. avium* 104. (**A**) ZF infected with *M. avium* 104 were treated with various concentrations of CHX (0.025, 0.05, and 0.1 μM) and CLA (50 μM). Subsequently, the bacterial burden in infected ZF was quantified using traditional agar plate quantification methods. Data represent the mean log10 CFU per embryo (*n* = 20 for each condition) from three independent experiments. Statistical analysis was determined with one-way ANOVA and presented as means ± SD. ***p < 0.001. (**B**) Survival of *M. avium* 104-infected embryos treated with 0.025, 0.05, and 0.1 μM CHX compared to CLA at 50 μM. Survival curves were compared with log-rank (Mantel-Cox) test. ns; not significant, *p < 0.05, ***p < 0.001, ****p < 0.0001. Inf UNT; infected untreated, CLA; clarithromycin.

For in vivo efficacy, ZF were infected with *M. avium* 104, and the efficacy of CHX was compared to that of CLA at concentrations of 0.025, 0.05, and 0.1 μM. To assess the therapeutic potential of CHX, we evaluated the in vivo efficacy in ZF infected with the *M. avium* 104 strain (Fig. 6). *In vivo* efficacy was determined using two approaches. First, after infection with *M. avium* 104 into ZF following CHX or CLA treatment, efficacy was quantified by CFU enumeration per ZF. A statistically significant reduction in bacterial load was observed at concentrations of 0.05 and 0.1μM after 5 days of treatment, indicating the bacterial growth inhibitory effect of CHX on *M. avium* 104 in ZF. Treatment with 0.1μM CHX led to an approximately 2.4 log10 CFU reduction on agar plates compared to the untreated control, comparable to CLA treatment at 50 μM (Fig. 6A). Secondly, we assessed whether higher concentrations of CHX could prolong the lifespan of *M. avium* 104-infected ZF compared to CLA. ZF survival was monitored using the Kaplan-Meier method for 13 days post-infection (dpi) after treatment with each drug. All ZF in the untreated group had died by 13 dpi. However, both CHX- and CLA-treated groups exhibited a concentration-dependent increase in ZF lifespan (Fig. 6B). When *M. avium* 104-infected ZF were exposed to 0.1 μM of CHX, their survival rates were 65%, comparable to CLA at 50 μM. In summary, these results demonstrate the therapeutic efficacy of CHX against *M. avium* 104 in an in vivo ZF model.

### Broad-Spectrum Antibacterial Activity of CHX against *Mycobacteria*l Species and Other Pathogenic Bacteria

To evaluate the potential antimicrobial activity of CHX, we assessed its efficacy against a range of bacterial species, including *M. tuberculosis*, *M. abscessus*, and representative Gram-positive and gram-negative pathogens. CHX exhibited activity against *M. tuberculosis* with an MIC_50_ value of 4.3 μM; however, no inhibitory effect was observed against *M. abscessus*. In addition, CHX was active against the gram-positive bacterium *Bacillus subtilis*, as well as the gram-negative bacteria *Escherichia coli* and *Salmonella enterica* subsp. *enterica* *serovar* Typhimurium. However, CHX showed no detectable activity against *Enterococcus faecalis*, *Staphylococcus aureus*, *Klebsiella pneumoniae*, or *Pseudomonas aeruginosa* at concentrations up to 25 μM.

## Discussion

*Mycobacterium avium*, predominantly associated with pulmonary infections, is also capable of causing skin diseases, albeit less frequently. Although most commonly encountered in patients with HIV (human immunodeficiency virus) infection, disseminated MAC with cutaneous lesions is becoming more common in patients receiving immunosuppressive medications. Cutaneous infections necessitate prolonged antibiotic regimens tailored to the drug susceptibility profile of the causative strain [20, 21]. The emergence of naturally resistant *M. avium* clinical isolates, particularly among immunocompromised patients, highlights the pressing need for novel antimycobacterial therapies [22]. To address this unmet need, we applied the Pandemic Response Box to identify potential drug candidates for *M. avium*. Using a resazurin-based dual screening assay targeting *M. avium* 104, we aimed to narrow down promising hits and advance the discovery of effective treatments for this pathogen. This particular strain was initially obtained from an AIDS patient's blood during routine diagnostic procedures in the laboratory [23]. From the screen, we identified five promising hits: erythromycin, clofazimine, eravacycline, bedaquiline, and AX, all exhibiting potent activity against *M. avium* 104 (Fig. 1). Erythromycin, a class of antibiotics that block the ribosome's polypeptide exit tunnel, inhibits translation [24]. Retrospective studies suggest that long-term, low-dose erythromycin monotherapy may potentially suppress the exacerbation of MAC-PD, with well-tolerated adverse events [25, 26]. Eravacycline, a fluorocycline approved for complicated intraabdominal infections in an intravenous formulation, disrupts bacterial protein synthesis [27]. Clofazimine, an FDA-approved lipophilic antibiotic for treating *Mycobacterium leprae*, inhibits mycobacterial respiratory chain and ion transporters, generating damaging reactive oxygen species [28]. Bedaquiline, the first anti-TB drug to impact bacterial energy metabolism, specifically inhibits mycobacterial ATP synthase, crucial for *M. tuberculosis* energy generation [29].

Lastly, this screening assay identified a potent bactericidal compound targeting MAC and designated as AX, which is chemically characterized as a bis-biguanide dihydrochloride (Fig. 2A). It was initially discovered owing to its potent antibacterial and antifungal activities against pathogens such as *Enterococcus faecalis*, *Candida albicans*, *Aspergillus fumigatus*, and *Cryptococcus neoformans* [30, 31]. Beyond its antimicrobial spectrum, AX is also known to induce mitochondrial dysfunction and apoptosis, indicating a broad bioactivity profile. Historically, AX has been employed as an antiseptic in mouthwashes and as a disinfectant in contact lens solutions. Notably, a 1%AX formulation has demonstrated significant efficacy in treating *E. faecalis* infections [32]. To further assess the activity of AX against *M. avium* 104, we included CHX, a well-characterized bis-biguanide antiseptic with structural and functional similarities to AX (Fig. 2B). Both compounds share a conserved bis-biguanide core that imparts strong cationic properties, facilitating electrostatic binding to negatively charged bacterial membranes. However, they differ significantly in their terminal hydrophobic moieties. AX contains two ethylhexyl groups, which enable deeper insertion into the lipid bilayer and rapid membrane disruption accompanied by lipid phase separation. In contrast, CHX carries two p-chlorophenyl groups and exerts bactericidal effects more gradually, primarily via electrostatic interactions that disrupt membrane phospholipids without inducing lipid domain separation [33]. These structural variations translate into distinct membrane-disruptive mechanisms, yet the shared bis-biguanide scaffold provided a rational basis for directly comparing their antimicrobial efficacy against *M. avium* in vitro, a comparison not previously documented. The distinct modes of action of AX and CHX were further reflected in our time-kill kinetics assays. AX exhibited markedly faster bactericidal activity than CHX, achieving complete eradication of *M. avium* within 24 h at 20× IC_50_, with no visible colony formation thereafter (Fig. 4). This rapid and concentration-dependent bactericidal effect is consistent with AX’s membrane-disruptive mechanism. In contrast, CHX showed a more gradual reduction in bacterial viability, with significant colony loss observed around 4 days post-treatment, particularly at higher concentrations. While slower in onset, CHX ultimately achieved levels of bacterial clearance comparable to AX. These results highlight the potent in vitro activity of both bis-biguanide compounds against *M. avium* and support their potential as fast-acting anti-NTM agents with distinct kinetic profiles.

Furthermore, our results (Table 1) demonstrate that both compounds exhibit potent bactericidal activity across multiple *M. avium* strains, including clinical isolates. The MIC_90_ values ranged from 0.8 to 2.1 μM, with MBCs between 0.8 and 3.1 for all tested strains. The MBC for each strain was found to be less than four times the MIC, indicating a characteristic feature of bactericidal antimicrobial agents [34]. This in vitro activity of AX and CHX was further studied in a ZF *in vivo* infection and treatment model. The ZF embryo infection model is a popular, tractable *in vivo* model that is often used to generate systemic infections for subsequent analysis, such as those in survival experiments and bacterial burden quantification [18]. This model has been extensively employed for assessing anti-bacterial drug efficacy against various mycobacterial species, including *M. abscessus* and *Mycobacterium marinum* [35-40]. Based on this simple and easy model, we evaluated the *in vivo* efficacy of AX and CHX by administering *M. avium* 104 via caudal vein injection into ZF. Due to the higher toxicity observed with AX in ZF MTD assays compared to CHX, only CHX was selected for further in vivo evaluation (Fig. 5A, 5B). Notably, the elevated toxicity of AX has been previously documented in macrophage-based assays, suggesting a broader cytotoxic potential that may limit its translational applicability [30, 41]. In contrast, CHX demonstrated considerable in vivo efficacy in the ZF infection model, significantly reducing bacterial CFUs in a dose-dependent manner compared to clarithromycin treatment (Fig. 6A). Survival analysis further revealed that while untreated *M. avium* 104-infected ZF exhibited 100% mortality by 11 dpi, treatment with as low as 0.1 μM CHX extended survival to approximately 65% (Fig. 6B). Importantly, previously reported cytotoxicity thresholds for CHX in RAW264.7 macrophages occur at concentrations exceeding 10 μM, with marked toxicity observed at ~20 μM [41]. The effective CHX concentrations used in this study were substantially lower—3 to 30-fold below these in vitro cytotoxic levels and approximately 200-fold below the in vivo toxicity threshold observed in ZF. These findings strongly support that the antimicrobial efficacy of CHX in both in vitro and in vivo settings is primarily attributable to its bactericidal activity rather than nonspecific cytotoxic effects. While further pharmacokinetic and pharmacodynamic (PK/PD) studies in mammalian models are necessary to confirm systemic applicability in humans, the observed ZF in vivo efficacy at submicromolar concentrations supports the potential for systemic therapeutic use. These findings provide proof-of-concept evidence that CHX can suppress systemic *M. avium* infections at doses significantly lower than known cytotoxic thresholds.

CHX has long been used as a topical antiseptic due to its broad-spectrum antimicrobial activity, particularly in oral and skin applications. However, its systemic use has been limited primarily because of potential toxicity, which can lead to hemolysis and nephrotoxicity at higher concentrations, as well as concerns regarding limited tissue penetration [42]. To date, CHX has not been explored as a systemic agent against MAC infections, likely due to these safety concerns and the lack of data supporting in vivo efficacy at tolerable doses. The present study demonstrates that CHX can exert potent antimycobacterial activity at submicromolar concentrations, suggesting that careful dose optimization could potentially overcome historical limitations and open new avenues for systemic or localized therapeutic applications against MAC infections.

In conclusion, this study highlights CHX as a promising, repurposed candidate for the treatment of *M. avium* infections, including potential systemic applications at submicromolar concentrations that minimize toxicity concerns. By demonstrating significant bactericidal activity in both *in vitro* and *in vivo* zebrafish systemic infection models, CHX overcomes historical barriers that have limited its systemic use. Although further PK/PD, safety, and efficacy studies in mammalian models remain necessary to confirm clinical applicability, our findings provide an important proof-of-concept foundation. Collectively, these results support the future development of CHX as a novel therapeutic option to address the growing clinical need for effective treatment against drug-resistant and extrapulmonary *M. avium* infections.

## Supplemental Materials

Supplementary data for this paper are available on-line only at http://jmb.or.kr.



## Figures and Tables

**Table 1:** Comparison of inhibitory potency of alexidine and chlorhexidine against *M. avium* reference strains and clinical isolates.

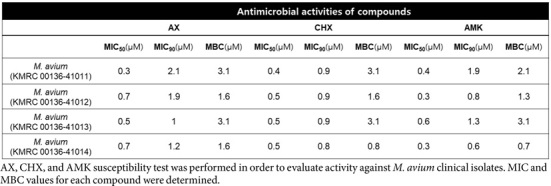

**Table 2:** Mutation frequency of AX and CHX compare to AMK.

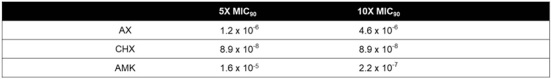

**Table 3:** Broad-spectrum antibacterial activity of CHX.