# Supplementary material for: Identification of a Drug Candidate against Mycobacterium avium Using Pandemic Response Box
Source: J Microbiol Biotechnol. 2025 Aug 18;35:e2506006. doi: 10.4014/jmb.2506.06006 (PMC12375541; doi:10.4014/jmb.2506.06006)
Supplement: Supplementary file 1 [file jmb-35-e2506006-supple.pdf]

J. Microbiol. Biotechnol. 2025. 35: e2506006
https://doi.org/10.4014/jmb.2506.06006

Supplementary Figure

A

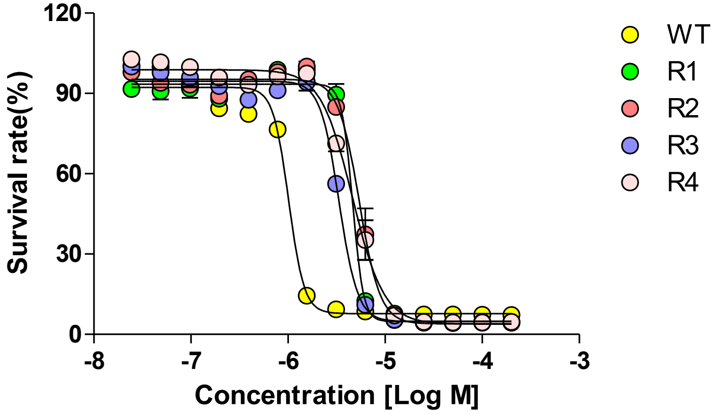

10x CHX	
	MIC_{50} (μM)
WT	1
R1	4.6
R2	5.4
R3	3.4
R4	4.6

B

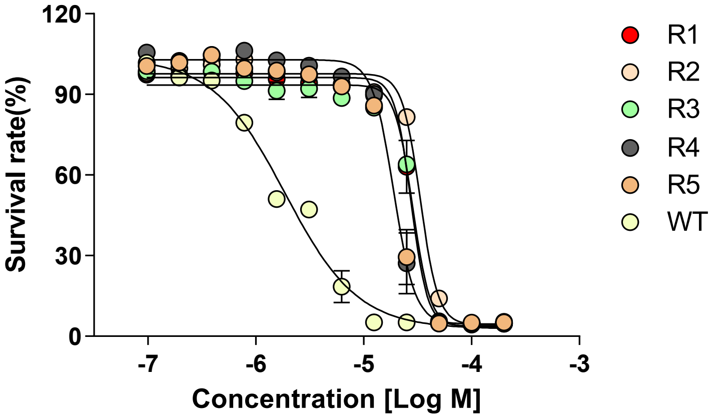

10x AX	
	MIC_{50} (μM)
WT	1.9
R1	27.6
R2	33.5
R3	28.4
R4	19
R5	19.3

Fig. S1. Drug response curves (DRC) and MIC values of CHX- and AX-resistant *M. avium* isolates.
(A) Dose-response curves of wild-type (WT) and CHX-resistant clones (R1–R4) treated with CHX. **(B)** Dose-response curves of WT and AX-resistant clones (R1–R5) treated with AX. Bacterial survival rates were determined following 4-days drug exposure. Error bars represent mean ± SD from at least three independent experiments. Corresponding MIC_{50} values for each isolate are summarized in the tables on the right.
